# Post-stroke spasticity: advances in network mechanisms and therapeutics

**DOI:** 10.3389/fnhum.2026.1868901

**Published:** 2026-09-24

**Authors:** Wei Yao, Yuanbin Yang, Zhiyi Wang, Longyue Yi, Deya Daneva, Zhuangwei Fang, Keyun Gan, Zhiqi Lin

**Affiliations:** 1 Department of First Rehabilitation Medicine, Wangjing Hospital of China Academy of Chinese Medical Sciences, Beijing, China; 2 School of Sports Medicine and Rehabilitation, Beijing Sport University, Beijing, China

**Keywords:** complication, network mechanisms, spasticity, stroke, therapeutics

## Abstract

Spasticity is one of the common complications of stroke, causing serious adverse effects for patients. However, comprehensive descriptions of the PSS network mechanism are currently lacking. This paper expounds on the mechanisms underlying post-stroke spasticity from multiple perspectives, including central nervous system networks, neurotransmitter networks, pain networks, and myofascial networks, as well as the brain-gut axis and brain-muscle axis networks. It summarizes emerging treatment approaches in recent years, providing a reference for clinical treatment and rehabilitation.

## 1 Introduction

Stroke is the third most prevalent cause of disability globally and the second greatest cause of death after ischemic heart disease, accounting for 11.8% of all fatalities (Feigin and Owolabi, 2023; He et al., 2022; Wu et al., 2019). China has the highest incidence of stroke worldwide, with 2 million new cases each year (Wu et al., 2019; Brainin, 2019). It is estimated that approximately one-third of stroke patients will experience a spastic state (Suputtitada et al., 2024). Patients may benefit somewhat from moderate improvements in muscle tone, such as when standing. However, increased muscular tone that causes spasticity has negative effects, such as pain and irregular movement patterns, which significantly reduce patients’ everyday functional abilities and place a financial and physical load on them.

One of the symptoms of upper motor neuron syndrome after damage to the central nervous system is spasticity, which is defined by velocity-dependently elevated muscular tone (Hwang et al., 2024). Conventional explanations center on injury to the central nervous system that results in excessive activation of the medial reticular spinal pathway, increased stretch reflexes, and altered peripheral muscle architecture. In recent years, neurotransmitters, pain, and the brain-gut axis have all been found to be closely associated with spasticity. Therefore, this paper reviews the pathological mechanisms of post-stroke spasticity from the above viewpoints and summarizes emerging treatment approaches in recent years, aiming to provide new insights for clinical management of spasticity, see Table 1 for a summary of the mechanisms.

**Table 1:** Summary of pathophysiological networks in PSS.

Network	Key components	Pathological changes after stroke
Central nervous system network	Cerebral cortex network	Excitatory-inhibitory imbalance between bilateral hemispheres
	Cortico-reticulospinal pathway	CRT damage and medial RST hyperexcitability
	Basal Ganglia-Thalamo-cortical	Disrupted connectivity
	Cerebello-Thalamo-cortical network	
	Spinal cord network	Presynaptic inhibition↓; Postsynaptic inhibition↓
Neurotransmitter network	GABA, Glu	Complex temporal changes in both neurotransmitters post-stroke
	5-HT, NE, DA	5-HT, NE↑, DA↓
Myofascial network	Myofascia	Hyaluronic acid ↑; myofascial thickening; MTrPs↑
Pain network	M1, ACC; SMN, SN, DMN	Disrupted connectivity among brain regions and networks
Gut-brain axis + Gut-muscle axis	Gut-Brain Axis:gut microbiota, BBB	LPS, TMAO↑, SCFAs↓; BBB disruption; systemic inflammation↑
	Gut-Muscle Axis:gut microbiota, skeletal muscle	Gut microbiota dysbiosis; Skeletal muscle spasticity (limited evidence)

CRT, Corticoreticular tract; RST, Reticulospinal tract; GABA, Gamma-aminobutyric acid; Glu, Glutamate; 5-HT, 5-Hydroxytryptamine; NE, Norepinephrine; DA, Dopamine; MTrPs, Myofascial trigger points; M1, Primary motor cortex; ACC, Anterior cingulate cortex; SMN, Sensorimotor network; SN, Salience network; DMN, Default mode network; BBB, Blood–brain barrier; LPS, Lipopolysaccharide; TMAO, Trimethylamine N-oxide; SCFAs, Short-chain fatty acids.

## 2 Central nervous system network

The functional abnormality of the central nervous system network is the core mechanism leading to post-stroke spasticity (PPS). After a stroke, several levels or structures in the network of the central nervous system experience direct or indirect injury, which together mediate the start and progression of spasticity through complex, interconnected neural pathway chains.

### 2.1 Cerebral cortex network

An imbalance between excitation and inhibition in both cerebral hemispheres, as well as aberrant functional connectivity among distinct brain regions, is caused by direct damage to brain tissue and indirect neural remodeling following a stroke. These factors are major causes of motor dysfunction, including spasticity (Li et al., 2019; Li et al., 2021; Krucoff et al., 2019) (Figure 1). In other words, stimulating the cortex of the affected hemisphere while inhibiting the cortex of the contralateral hemisphere is the key to alleviating spasticity from a cortical perspective. When treating elderly stroke patients with brain-computer interface functional electrical stimulation (BCI-FES), Chen et al. found that the theta-band power at the Cz and C4 electrodes and the alpha-band power at the FC3 and CP3 electrodes dramatically increased after therapy. This implies that improvements in motor function were substantially linked with an increase in theta-band power at the Cz electrode, and that BCI-FES may have its therapeutic impact by encouraging functional reorganization of the contralateral cerebral hemispheres (Chen et al., 2025). To activate the primary motor cortex(M1) in the affected hemisphere, Wang used 10 Hz repetitive transcranial magnetic stimulation(rTMS), and Chen used 1 Hz rTMS to inhibit M1 in the contralateral hemisphere. They promoted neural remodeling and alleviated spasticity by both directly and indirectly increasing excitability in the M1 of the affected hemisphere (Wang et al., 2025; Chen et al., 2023). Spasticity is also strongly linked to inadequate excitability in the affected hemisphere’s supplementary motor area (SMA). Increased activation levels in the affected side’s SMA have been linked to reductions in spasticity in PSS patients, according to research. This can improve facilitative inputs to descending inhibitory pathways (Qin et al., 2023). PSS is not only associated with dysfunction in motor-related brain regions but also exhibits significant connections with sensory-related cortices. According to an imaging study, PSS patients had significantly less resting-state functional connectivity between the superior parietal lobule in the contralateral hemisphere and the intraparietal sulcus in the parietal cortex of the affected hemisphere than non-spastic stroke survivors. Furthermore, the severity of the spasticity increases with decreasing connection. Increased hemisphere connectivity was noted after receiving injections of botulinum toxin, and higher connectivity was associated with more noticeable improvements in spasticity (Veverka et al., 2023). The perception-motor feedback loop may be disrupted by weakening transhemispheric connections in the parietal cortex, which can result in aberrant motor control and exacerbate spasticity. Furthermore, another study discovered that after receiving focal vibration, PSS patients had improved connectivity in both hemispheres of the primary somatosensory cortex (S1) and M1. Ipsilateral S1-M1 connection increased more than contralateral S1-M1 connectivity, though. This implies that an imbalance in S1-M1 activity in both cerebral hemispheres may be linked to PSS (Li et al., 2022). According to research on the structure of the cerebral cortex, alleviation of PSS is associated with thicker sensorimotor cortex tissue. This suggests that post-stroke spasticity is strongly linked to structural remodeling in the brain in addition to being caused by decreased functional connectivity across several cortical regions (Hurtado-Olmo et al., 2025).

**Figure 1:**
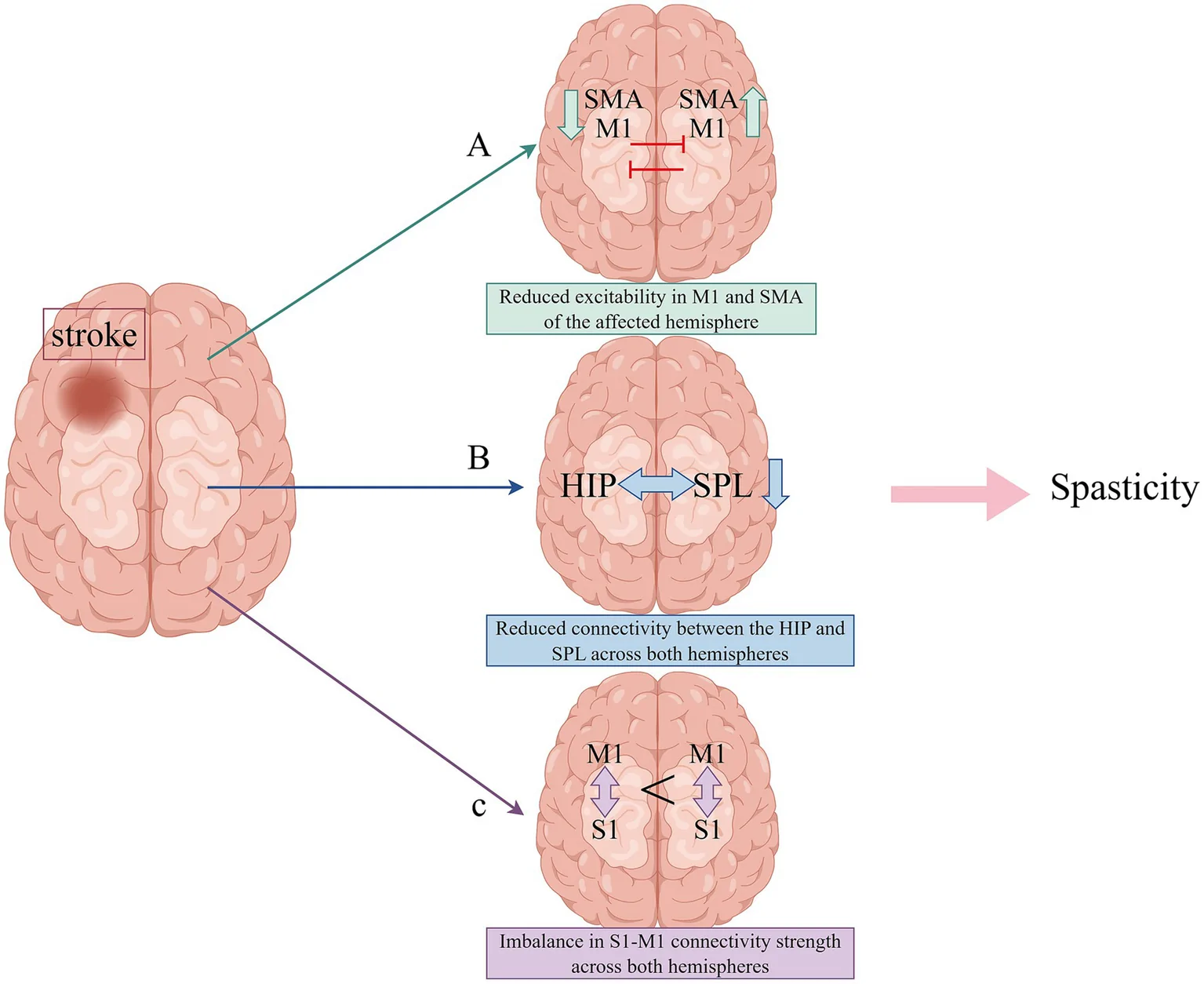
Spasticity is linked to abnormal functional connectivity in the cerebral cortex after stroke. **(A)** Increased excitability in the contralateral hemisphere and decreased excitability in the affected hemisphere’s M1 and SMA. **(B)** Decreased connectivity strength between the affected hip region and the contralateral SPL after stroke; **(C)** Imbalanced connectivity strength between S1 and M1 across both hemispheres, with weaker connections on the affected side compared to the contralateral side. SMA, Supplementary motor area; M1, Primary motor cortex; IPS, Intraparietal sulcus; SPL, Superior parietal lobule; S1, Primary somatosensory cortex. This picture was drawn by Figdraw.

### 2.2 Cortico-reticulospinal pathway

The corticoreticular-spinal pathway comprises the corticoreticular tract (CRT) and the reticulospinal tract (RST). CRT originates from the premotor cortex (PMC) and SMA in the cerebral cortex and terminates in the pontomedullary reticular formation (PMRF) (Azizi et al., 2024; Jang et al., 2013). The RST, which is made up of the dorsal and medial columns, is another important descending fiber tract besides the corticospinal tract (CST). The medial RST primarily originates from the pontine tegmentum, receiving excitatory inputs from the ipsilateral PMC, SMA, and M1, and providing excitatory inputs to spinal motor neurons. This excitatory signal is primarily mediated by monoamine neurotransmitters such as serotonin and norepinephrine (Li et al., 2019). The ventromedial reticular formation in the medulla oblongata is the primary source of the dorsal RST, which receives facilitative input from the contralateral M1 through the CRT and gives spinal motor neurons inhibitory input (Li et al., 2019). The spinal motor neuron network’s excitatory and inhibitory forces are dynamically balanced by the RST’s medial and dorsal columns (Figure 2). The CRT is located anatomically beneath the cortex, passing through the centrum semiovale, corona radiata, and posterior limb of the internal capsule before arriving at the midbrain’s tegmentum, reticular formation of the pons, and reticular formation of the medulla (Jang and Lee, 2019). The “bridge” between the motor cortex and the inhibitory area of the medullary reticular structure is damaged when a stroke is accompanied by damage to the cortex or internal capsule. This results in a lack of inhibitory input to counteract the medial RST, which causes excessive excitation of spinal motor neurons. This is thought to be the most fundamental mechanism of spasticity following stroke. Additionally, in a photochemically induced post-stroke spasticity mouse model, recent research by Isumi et al. using quantitative activation-induced manganese-enhanced MRI technology found that neuronal activity in the contralateral ventral medullary reticular formation (MdV) significantly increases during the acute phase post-stroke, and that excessive excitation of MdV neurons activates neural circuits associated with spasticity. Immunohistochemical staining also revealed that the density of AMPAR subunit GluA1 on the synapses of MdV neurons significantly increased during the acute phase, and the GluA1/GluA2 ratio changed 2 weeks post-stroke, indicating that homeostatic plasticity is involved in the occurrence of PSS (Isumi et al., 2024). The brainstem is a crucial interface between the upper motor centers and spinal cord circuits. This study indicates that changes in the excitability of MdV neurons after a stroke also play an important role in the occurrence and development of PSS.

**Figure 2:**
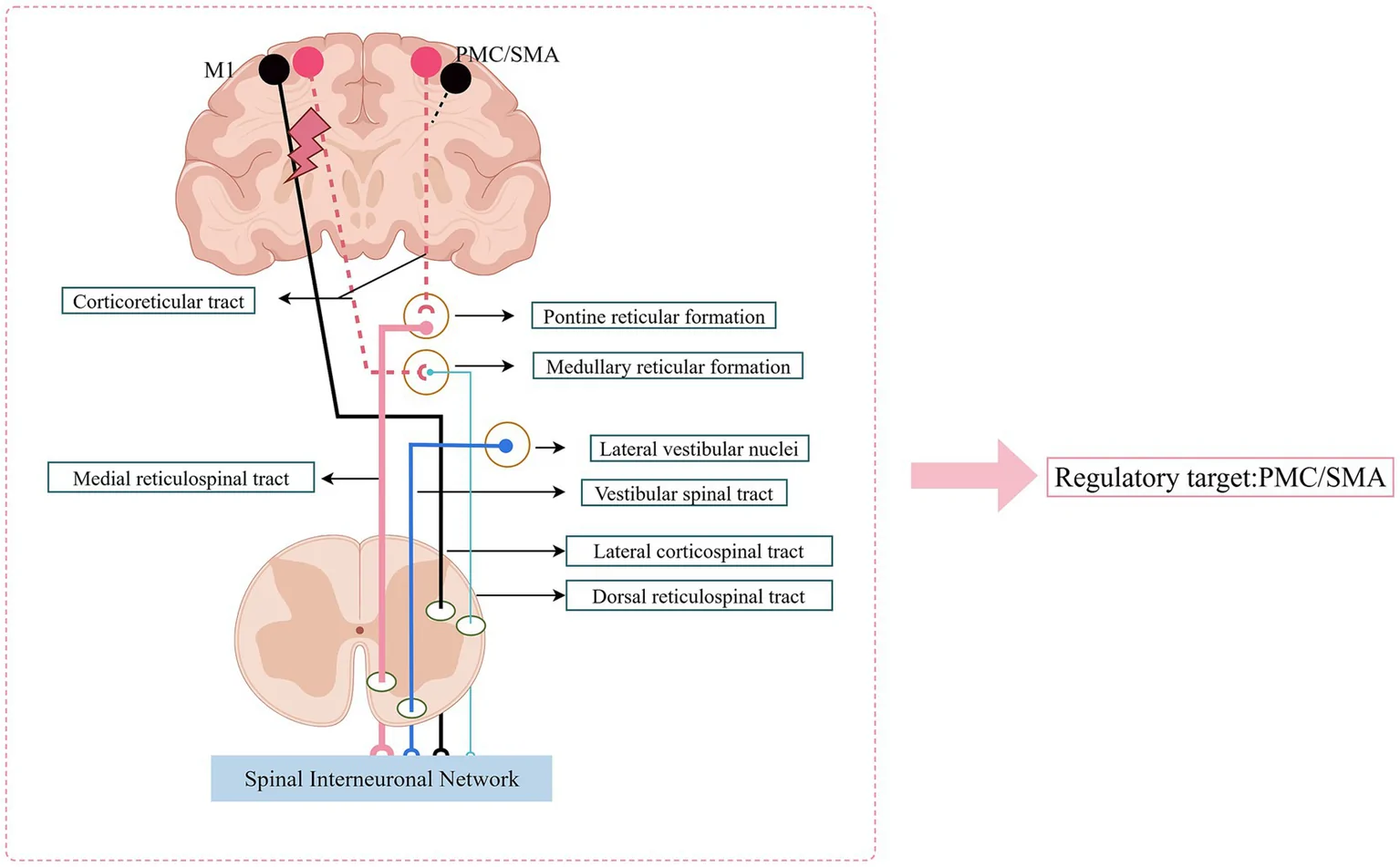
Aberrant cortico-reticulospinal pathway after stroke. The loss of cortical facilitation resulting from stroke damage to the CRT weakens the inhibition of the dorsal RST. Due to a lack of balanced inhibitory control, the medial RST causes stretch reflexes that overexcite the spinal network. This picture was drawn by Figdraw.

There is an ongoing debate on the vestibulospinal tract’s(VST) involvement in PSS. Animal research does not suggest a role for the VST in spasticity (Li et al., 2019), while human studies have shown a substantial association between trigger responses and spasticity severity (Miller et al., 2016).

Inhibiting the PMC, SMA, and M1 of the contralateral hemisphere in PSS patients may be a useful treatment strategy because these brain areas supply excitatory input to the medial RST via CRT. Although M1 is a commonly used target for non-invasive neuromodulation in clinical practice, different studies have found different levels of success with this treatment for spasticity. PMC-derived neural fibers are essential for controlling the tone of limb muscles. Therefore, Zhang et al. chose the PMC in the contralateral hemisphere as the stimulation target. Significant improvements in spasticity were seen in PSS patients following 2 weeks of combined continuous theta burst stimulation (cTBS) and acupuncture intervention. The effect size changes were greater than the minimal clinically important difference, indicating clinical significance (Zhang et al., 2025). Similarly, future studies can explore whether SMA as a target for regulating PSS is equally effective for spasm.

### 2.3 Basal ganglia-thalamo-cortical and cerebello-thalamo-cortical networks

Damage to the basal ganglia increases the risk of developing spasticity, according to research on the association between lesion site and spasticity occurrence in stroke patients (Zhu et al., 2024). The thalamus, cerebral cortex, cerebellum, and other structures are all closely connected to the basal ganglia by neural networks. Key lesion locations in PSS have been studied in a recent voxel-based lesion-symptom mapping study. Two key lesion clusters were eventually identified after the study extracted 55 lesions for meta-analysis from 172 stroke spasm patients. Both clusters were part of the basal ganglia-thalamo-cortical network, with one cluster mostly found in the thalamus and the other in the globus pallidus (Favre et al., 2025). Both lesion clusters were shown to be primarily related to the S1 (accounting for 53.8% of all nerve fibers), followed by M1 (15.9%), according to structural connectivity network analysis (Favre et al., 2025). This suggests that PSS is closely linked to the sensorimotor cortex and linked to harm to the basal ganglia-thalamo-cortical network.

Additionally, spasticity is closely related to the cerebello-thalamo-cortical network. Multiple randomized controlled trials have found that neuromodulation techniques targeting the cerebellum can significantly improve spasticity in stroke patients. Chen et al. applied intermittent theta burst stimulation (iTBS) to the affected hemisphere’s cerebellar vermis in subacute patients (Chen et al., 2021), and Li et al. chose to use low-frequency rTMS of the contralateral M1 in conjunction with cTBS of the right cerebellum (Li et al., 2021). Another study examined the effect of spasticity improvement after receiving low-frequency rTMS to the contralateral M1 and iTBS to the contralateral cerebellum, as well as low-frequency rTMS to the contralateral M1 and cTBS to the affected cerebellum (Chen et al., 2025). Together, the results of the previously described research show that reducing spasticity can be achieved by specifically targeting the cerebellum as a neuromodulation site. Interestingly, the outcomes consistently showed that stroke patients’ spasticity had improved, even though the different research methodologies varied. Although the exact pathway is still unknown, the cerebello-thalamo-cortical network may be involved in the assumed mechanism. According to Chen et al., the cerebellum integrates multimodal information from proprioception, vestibular input, and vision to dynamically modulate cortical motor area excitability. It also regulates synaptic plasticity in M1 through projections from the dentate nucleus to the ventrolateral nucleus of the thalamus (Chen et al., 2025).

Although the basal ganglia-thalamo-cortical and cerebello-thalamo-cortical networks are two distinct pathways, the thalamus appears to serve as a “bridge.” Input signals from the cerebellum and basal ganglia exhibit overlapping patterns in the thalamus. Therefore, Favre et al. hypothesize that stimulating the cerebellum may improve spasticity by resetting the functional imbalance between the basal ganglia-thalamo-cortical and cerebello-thalamo-cortical networks (Favre et al., 2025).

### 2.4 Spinal cord network

The intrinsic neuronal conduction network of the spinal cord also experiences secondary changes after stroke. Excessive excitation of spinal alpha motor neurons is the core mechanism underlying hyperactive stretch reflexes. Actually, the spinal cord contains multiple inhibitory neuronal circuits to prevent excessive stretch reflexes from injuring muscles (Figure 3). However, these spinal cord inhibitory neuronal pathways show varied degrees of abnormal attenuation after stroke.

**Figure 3:**
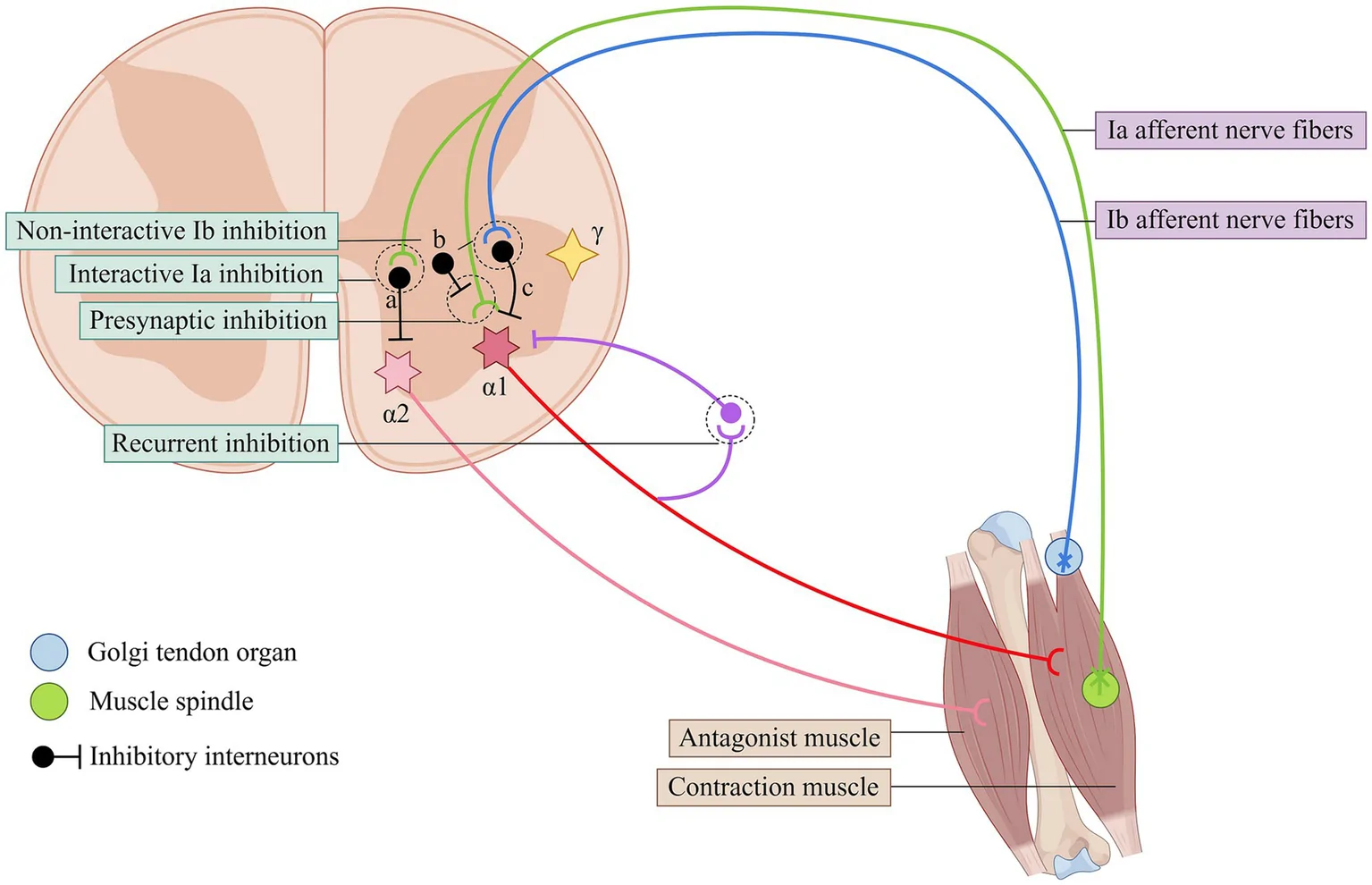
Inhibitory pathways associated with the stretch reflex in the spinal network. **(a)** Ia inhibitory interneurons; **(b)** Presynaptic inhibitory interneuron; **(c)** Ib inhibitory interneurons. This picture was drawn by Figdraw.

According to research, stroke patients’ paretic limbs show longer durations of excitatory postsynaptic potentials (EPSPs) at the Ia afferent fiber-α motor neuron synapse than do the unaffected limb and healthy persons. With α motor neurons remaining depolarized for a long time, this suggests slower EPSPs decay and increases the excitability of motor neurons fundamentally (Son et al., 2019). The underlying cause may be the attenuation of presynaptic inhibition and post-activation inhibition following stroke. If we delve deeper into why presynaptic inhibition and post-activation inhibition diminish, an animal study appears to provide an answer. Takuya and colleagues found that the inhibitory effects of γ-aminobutyric acid (GABA) and glycine were impaired on motor neurons in the spinal cord layer of PSS mice due to a significant reduction in potassium-chloride cotransporter 2 expression (Toda et al., 2014). Among these, KCC2 is a crucial transporter on the membranes of mature neurons that actively pumps intracellular Cl^−^ out of the cell. Its function is to keep intracellular chloride concentration low, which is a necessary precondition for the hyperpolarization brought on by the two main inhibitory neurotransmitters, glycine and GABA (Cherubini et al., 2021; Talifu et al., 2022). In a mouse stroke model, Toda et al. discovered that 7 days after the stroke, KCC2 expression was markedly downregulated in spinal motor neurons and that the dephosphorylation of the Ser940 site caused an imbalance in chloride homeostasis (Toda et al., 2014). Cl^−^ builds up intracellularly in spinal motor neurons when KCC2 expression is downregulated. This depolarizing shift in the Cl^−^ equilibrium potential causes GABA- and glycine-mediated inhibitory postsynaptic potentials to become depolarizing potentials or even to have excitatory effects. Moreover, the effectiveness of certain antispasmodic therapies is linked to the restoration of KCC2 function. For instance, acupuncture can increase KCC2 expression via modifying the NMDAR-PP1/Calpain1-KCC2 pathway, which prevents excessive stimulation of spinal cord neurons (He et al., 2025). On the other hand, exercise-induced head needles reduce post-stroke spasticity by reactivating KCC2 via 5-HT2AR (Zhang et al., 2025). Thus, one of the main mechanisms underpinning the development of post-stroke spasticity is the increased neuronal excitability brought on by decreased KCC2 function following a stroke.

However, the attenuation of inhibition following activation is more likely attributed to intrinsic abnormal remodeling of the spinal cord after stroke (Yang et al., 2015). Additionally, dysregulation of the non-reciprocal Ib inhibitory action of the Golgi tendon organ may also contribute to the onset of spasms (Morita et al., 2006). However, conflicting results exist regarding the roles of interactive Ia inhibition and return inhibition in the PSS, leaving their effects on spasm still uncertain (Bhagchandani and Schindler-Ivens, 2012; Chen et al., 2025). Furthermore, the study discovered a significant increase in the number of excitatory Ia afferent fibers influencing spinal motor neurons, which could indicate abnormal spinal cord remodeling (Toda et al., 2014). This also implies that the excitatory effects on Ia afferent motor neurons are enhanced after stroke, leading to hyperactive stretch reflexes. The role of enhanced excitability in gamma motor neurons in the onset and progression of spasticity remains to be determined and requires further investigation in future studies (Li and Francisco, 2015).

It is important to note that research has shown that after a stroke, damage to the corticospinal tract increases compensatory dependence on indirect motor pathways, especially the reticulospinal pathway, and that this compensation is an example of maladaptive plasticity. In contrast to the corticospinal tract’s direct monosynaptic transmission, indirect pathways entail multisynaptic interactions that result in longer time delays and increased nonlinear distortion of signal transmission. These factors ultimately cause a sustained increase in excitatory input to spinal α-motor neurons (Yang et al., 2020). Maladaptive synaptic remodeling is one of the main causes of neuronal hyperexcitability, which is a crucial component in the development of PSS.

## 3 Neurotransmitter network

The primary cause of central nervous system dysfunction after a stroke is abnormal alterations in neurotransmitters at neuronal synapses. Existing research shows the importance of abnormal neurotransmitter changes in PSS, even though the majority of current studies concentrate on changes in neurotransmitters and their corresponding mechanisms after cerebral infarction or hemorrhage, with few studies explicitly addressing PSS.

### 3.1 Amino acid neurotransmitters

Glutamate (Glu) and Gamma-Aminobutyric Acid (GABA) are the primary excitatory and inhibitory neurotransmitters in the central nervous system, respectively. Notably, these two neurotransmitters undergo dynamic and complex changes after a stroke, and they both have different roles in functional recovery at different stages of the stroke process (Figure 4). Therefore, we primarily review how Glu and GABA change over time following stroke and their relationship to the occurrence of spasticity.

**Figure 4:**
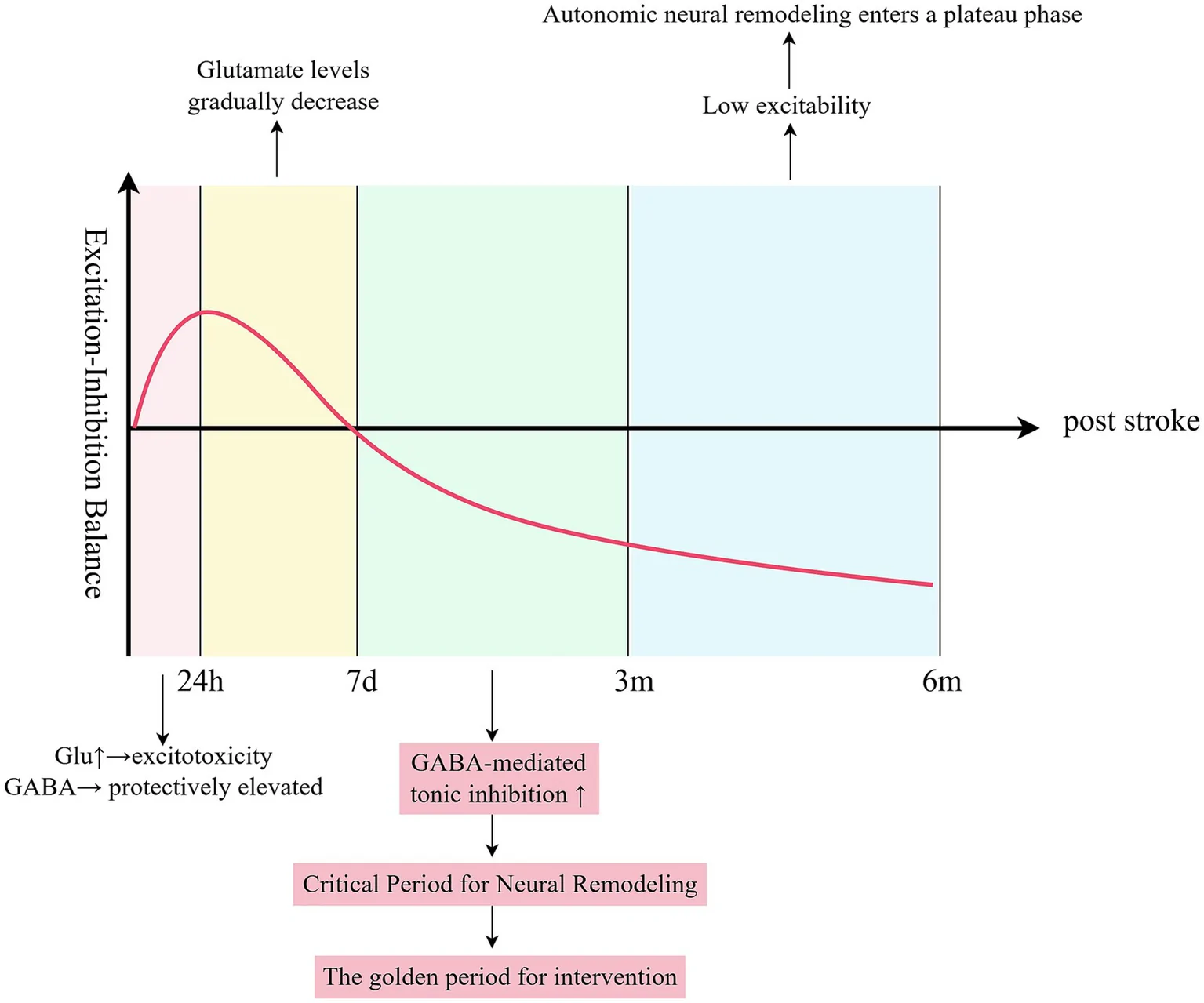
Time course of excitatory-inhibitory balance changes in the affected cerebral hemisphere after stroke. Within 24 h, Glu-mediated excitotoxicity predominates, accompanied by a protective increase in GABA; subsequently, glutamate levels gradually decrease while GABA-mediated tonic inhibition progressively strengthens. This hinders both neural reconfiguration and functional recovery by causing reduced excitability in the affected hemisphere during the subacute phase, which is a crucial time for neural remodeling. In the chronic phase, the affected hemisphere remains hyperexcitable, with spontaneous neural remodeling having reached a plateau. PSS arises from this abnormal neural remodeling. This picture was drawn by Figdraw.

According to the Rehabilitation Roundtable Consensus, the post-stroke period is categorized as follows: the hyperacute phase within 24 h, the acute phase from 1 to 7 days, the early subacute phase from 7 days to 3 months, the late subacute phase from 3 to 6 months, and the chronic phase beyond 6 months (Bernhardt et al., 2017). The concentration gradient collapses in the hyperacute phase due to acute ischemia and hypoxia in injured brain tissue. Excitotoxicity results from the massive release of Glu from presynaptic membranes and poor astrocyte reuptake, which causes the Glu concentration to rise quickly to 10–100 times physiological levels. Multiple brain networks become dysfunctional as a result of the widespread neuronal death that occurs within the lesion core (Lai et al., 2024). The damaged side’s cerebral cortex’s GABA content typically rose over the course of 24 h, with animal research showing a peak within 3 h (Ruan et al., 2017). This could be a protective elevation in which phasic inhibition mediated by γ-aminobutyric acid sub-type A receptors (GABAARs) on the postsynaptic membrane counteracts glutamate-induced excitotoxicity. However, due to the severity and complexity of stroke patients’ conditions during the hyperacute phase, clinical research on this phase remains limited. Both glutamate and GABA exhibit complex and irregular changes over time. Studies indicate that serum glutamate levels may gradually decrease 24 h after stroke onset, but some patients exhibit persistent elevation. Those with sustained elevation demonstrate poorer recovery of motor function (Yang et al., 2005). Subsequent GABA changes were much more complex, exhibiting contradictory phenomena. Animal research shows that extrasynaptic GABAARs in the peri-infarct area induce enhanced tonic inhibition throughout the acute and early subacute stages, which can last for more than a month (Clarkson et al., 2010). Additionally, clinical research has shown that subacute stroke patients’ affected M1 exhibits considerably higher levels of short-interval intracortical inhibition (SICI_1_ms), which shifts the excitatory-inhibitory balance towards inhibition (Cirillo et al., 2020). Neural remodeling is most important during the first 3 months after a stroke. Normal neuronal remodeling and signal transmission cannot be maintained in the affected hemisphere due to decreased excitability. A major contributing factor to abnormal neuronal remodeling, which is thought to be a crucial mechanism behind the onset of PSS, is the imbalance between excitation and inhibition during this time (Li et al., 2019; Li et al., 2021; Krucoff et al., 2019). Paradoxically, studies have found that stroke patients exhibit lower GABA levels at the 3-month mark compared to healthy controls, and this reduction may even persist into the chronic phase (Kim et al., 2014). During the chronic phase, the direct or indirect objectives of multiple clinical studies on neuromodulation techniques are to enhance excitability in the affected hemisphere, thereby improving motor function and reducing spasticity (Kuzu et al., 2021; Barros Galvão et al., 2014; Bernal-Jiménez et al., 2024). In conclusion, after a stroke, Glu and GABA experience complex and dynamic oscillations that cause a dynamic shift in the excitatory-inhibitory balance in the damaged hemisphere. It is yet unknown what fundamental processes underlie the intricate changes in GABA that occur during the subacute and chronic stages. Nonetheless, motor recovery seems to be enhanced by specifically blocking extrasynaptic GABAAR-mediated tonic inhibition. The objective of the subacute and chronic phases is to promote normal neural remodeling patterns, improve descending inhibitory control over the dorsal RST, and increase excitability in the affected hemisphere, all of which aid in the rehabilitation of spasticity and the restoration of motor function.

### 3.2 Monoamine neurotransmitters

PSS is also strongly linked to monoamine neurotransmitters. The monoamine neurotransmitters serotonin (5-HT) and norepinephrine (NE) are the main mediators of the excitatory input signals that come from the descending medial RST of the PMRF (Li et al., 2019). Among these, 5-HT increases persistent inward currents and increases the excitability of spinal motor neurons. According to earlier research, finger flexor spasm is linked to increased 5-HT release after a stroke and disinhibition of brainstem pathways (Seo et al., 2011). NE primarily increases muscle tone by amplifying the excitatory effects of Glu (Schwarz et al., 2008). Dopamine(DA) levels in the infarcted motor cortex of mice in a stroke-induced spasticity model were found to be significantly lower in animal experiments; however, after acupuncture therapy, DA levels were found to dramatically increase and spasticity improved (Zhang et al., 2023). However, compared to a placebo, exogenous levodopa supplementation did not significantly improve motor function recovery, according to a recent multicenter clinical research (Engelter et al., 2025). This could be because levodopa fails to specifically target the right receptors, acts non-specifically on different or even opposing dopaminergic systems, or damages the dopaminergic system during the acute phase, resulting in downregulated receptor expression and an insufficient response to exogenous drugs (Engelter et al., 2025). Furthermore, unlike Parkinson’s disease, stroke is characterized by extensive damage to the brain’s neuronal networks; hence, levodopa supplementation alone might not produce the desired results. Therefore, it seems that we cannot just concentrate on DA levels when examining its function in spasticity; we also need to look at changes in the expression of its different receptors after a stroke.

## 4 Myofascial network

The body’s muscles, bones, blood arteries, and nerves are all surrounded by a three-dimensional network of connective tissue called the myofascial network. Multiple myofascial lines are formed by this vast network of collagen fibers that connect muscles and surrounding soft tissues. It is a critical communicator between the neurological and musculoskeletal systems that spans the entire body. It is essential for maintaining proper muscle tone as well as for transmitting mechanical information (Schoenrock et al., 2018). The key characteristic of the myofascial network is continuity, which emphasizes that each muscle of the human body is interconnected through the myofascial network and functions as an organic whole. This also means that if a muscle has a problem, it will affect the distant muscles through the myofascial network and break the mechanical balance. As a vital peripheral tissue, increasing research has recently highlighted the myofascial network’s crucial peripheral role in PSS.

Choi and his colleagues recently found that the myofascial tissues on the front, rear, and sides of the affected leg were around 30% thicker than those on the unaffected side in individuals with chronic stroke spasticity. The ankle joint’s range of motion for plantar flexion and dorsiflexion was severely limited as a result (Choi et al., 2024). Elaine et al. also found that patients with lower limb spasticity after stroke had a substantial thickening of the gastrocnemius muscle fascia (Magat et al., 2025). Similarly, another study has reported that muscle shortening following stroke, caused by thickened myofascia impeding muscle fiber gliding, primarily occurs in muscles with extensive myofascial tissue, such as the pectoralis major, biceps brachii, and pronator teres. Furthermore, the myofascial network connecting these muscles results in the characteristic abnormal flexor synergistic posture of the upper limb, characterized by shoulder internal rotation, elbow flexion, and forearm pronation (Raghavan et al., 2016).

The increased viscosity of the extracellular matrix brought on by increasing hyaluronic acid levels is one of the main causes of anomalies in the myofascial network after stroke. This viscosity prevents muscle fibers from moving against the myofascial tissue, which results in myofascial thickening and fibrosis, muscle shortening and stiffness, and ultimately limited joint movement (Choi et al., 2024). Resistance during passive movement is increased by this mechanism (Figure 5). Based on myofascial chain theory, Raghavan et al. injected recombinant hyaluronidase solution at a maximal dose of 600 IU into many muscles of the spastic upper limbs in patients with chronic PSS, including the pronator teres, biceps brachii, and pectoralis minor. After 2 weeks of treatment, the results demonstrated a significant increase in passive range of motion for wrist, elbow, and shoulder flexion/extension. The improvements persisted at the three-month follow-up. The greater efficacy of injections along myofascial chains suggests that chronic PSS is significantly influenced by hyaluronic acid-induced myofascial network dysfunction (Raghavan et al., 2016). It has also been demonstrated that an elevation in acetylcholine at the motor endplate nerve terminals of muscles with myofascial trigger points (MTrPs), as well as the pain elicited by MTrPs, results in heightened muscle tension (Ma et al., 2020). In a multicenter clinical study with 210 participants, those who had chronic hand spasticity after stroke were treated by stimulating myofascial trigger points with dry needling. After 4 weeks of treatment, the results demonstrated significant reductions in spasticity levels in the wrist and all five fingers, with higher efficacy when compared to the non-myofascial trigger point dry needling group (Zhang et al., 2021). Acupuncture may work by successfully breaking the vicious cycle of spasm-pain-spasm at myofascial trigger points, which decreases muscular tone. Furthermore, studies have shown that manual release techniques that target the superficial dorsal pathway, one of the Myofascial Meridians, and acupoint selection based on the theory of myofascial meridians for acupressure therapy can effectively reduce limb spasticity after stroke (Pan et al., 2024; Jiang et al., 2024). This emphasizes even more how important the myofascial network is in PSS.

**Figure 5:**
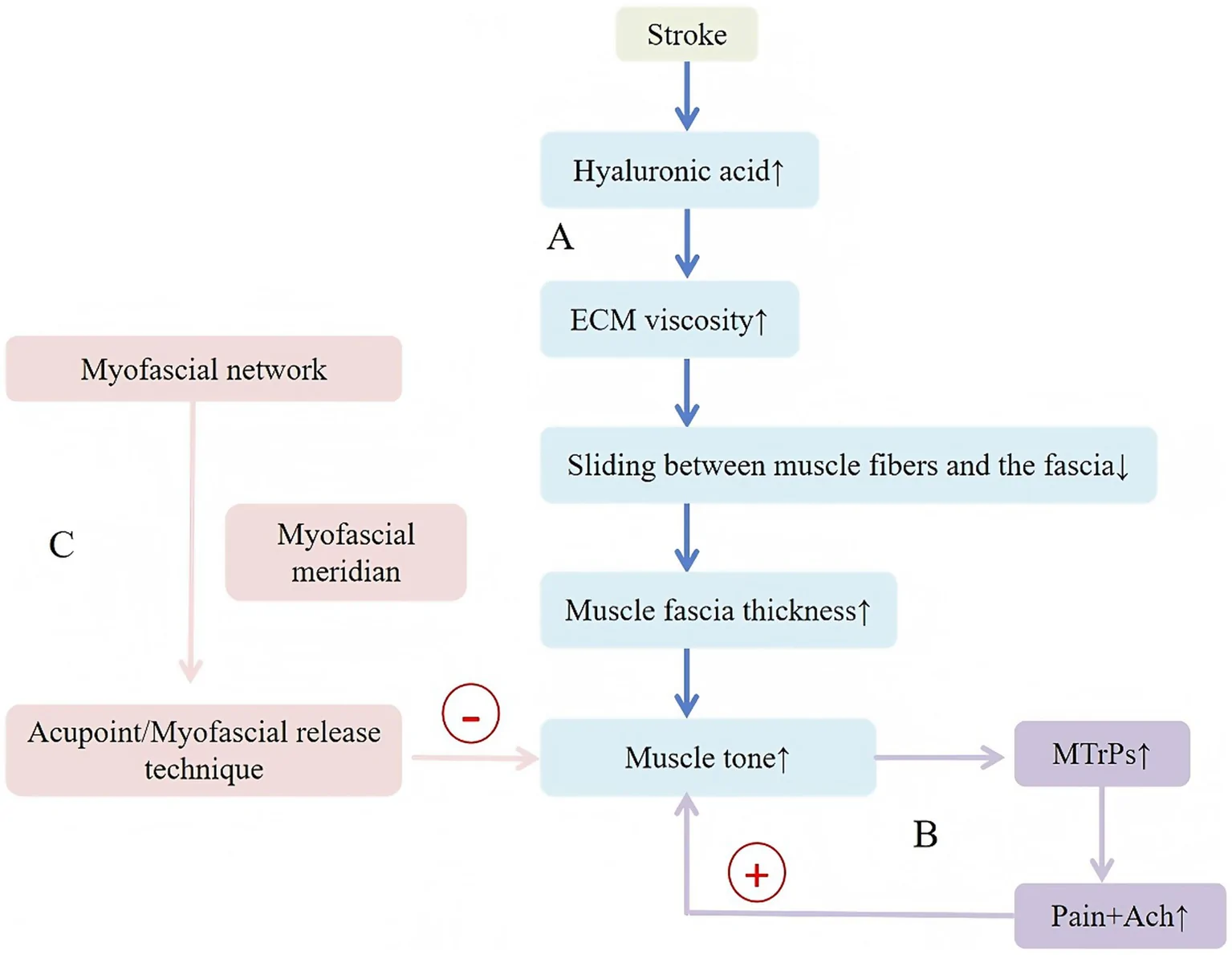
Schematic diagram of the myofascial network. **(A)** Hyaluronic acid levels increase in peripheral muscles following stroke, leading to myofascial thickening and fibrosis, which increases resistance during passive joint movement; **(B)** Elevated muscle tone activates myofascial trigger points (MTrPs), causing pain and increased acetylcholine (Ach) at neuromuscular junctions, further exacerbating muscle tension; **(C)** Based on myofascial chain theory, acupoint therapy and manual release techniques have been found to improve excessive muscle tone.

## 5 Pain network

Clinically, it can be found that PSS patients have a higher incidence of pain and a lower quality of life (Shaikh et al., 2016). The relationship between pain and spasm remains unclear. The relationship between the two seems not to be a simple one-way arrow, but may be a two-way complex interaction, or may be a parallel relationship. Existing research evidence indicates that the brain network associated with post-stroke pain overlaps with the PSS-related brain network (Figure 6).

**Figure 6:**
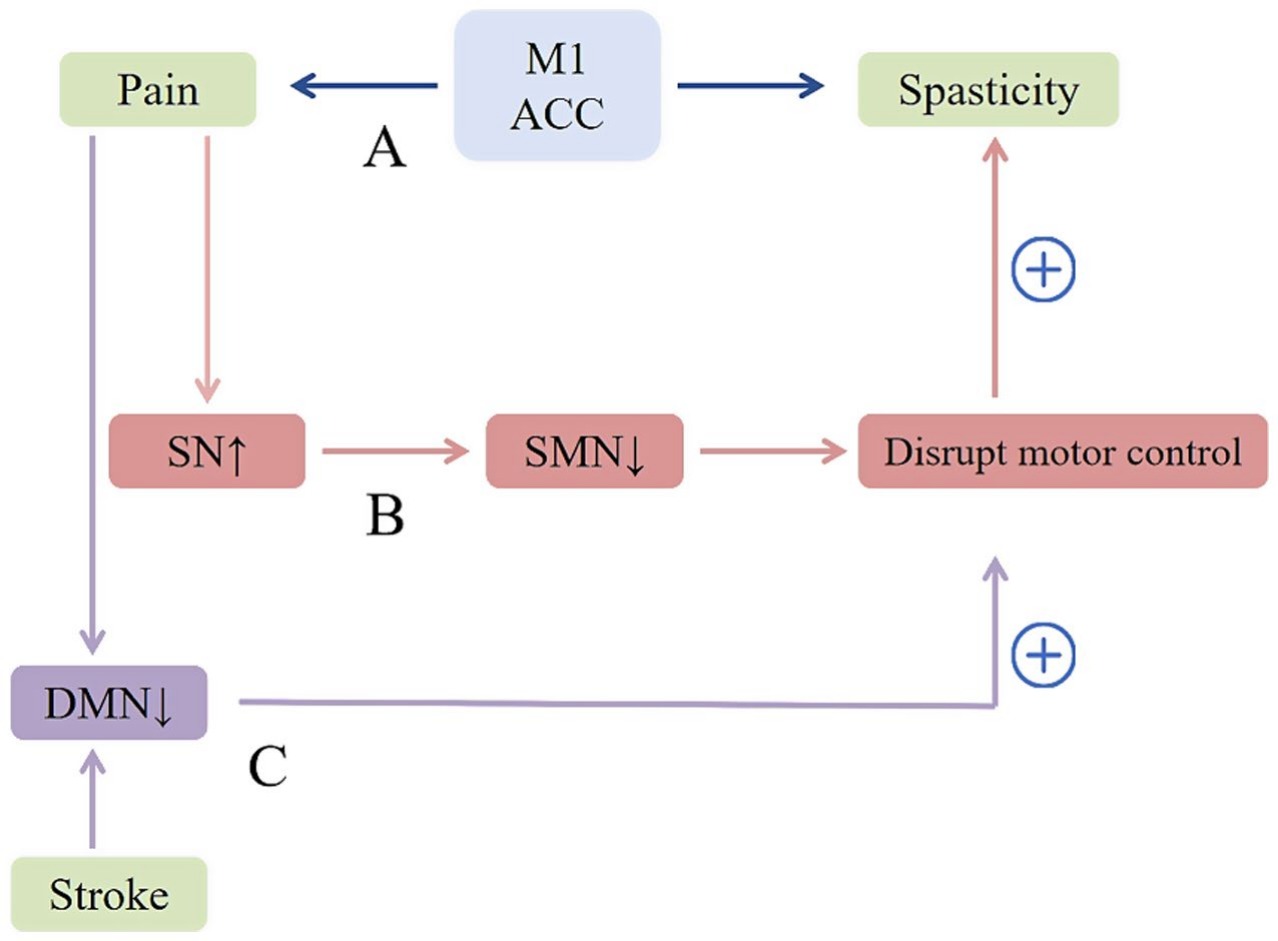
Schematic diagram of pain networks. **(A)** Primary motor cortex M1 and anterior cingulate cortex ACC represent overlapping brain regions for pain and spasticity. **(B)** Pain activates the SN, leading to reduced SMN activation, disrupting motor control, and promoting spasticity. **(C)** Both pain and stroke reduce DMN activation, thereby disrupting motor control, and promoting spasticity.

The processing of pain involves the coordinated interaction of multiple brain regions, including the thalamus, insula, somatosensory cortex, anterior cingulate cortex, dorsolateral prefrontal cortex, and M1 (Elias et al., 2020). This implies that a complex pain neural network is formed by the serial or parallel connections of several neural pathways in the central nervous system. Within M1, cortical activation responsible for both sensory input and motor output was observed alongside direction-specific functional connectivity (Huber et al., 2017). Clinical practice has further demonstrated that M1 serves not only as a traditional neural modulation target for PSS but also as a prominent brain region for pain treatment. The neural mechanisms underlying pain regulation through modulation of the M1 region may involve suppressing thalamic activity and enhancing activity in the periaqueductal gray, which, respectively, relate to inhibiting ascending inputs from nociceptive stimuli and promoting descending inhibitory pathways for pain (Gan et al., 2022). Additionally, Mayorova et al. used fMRI to observe changes in functional connectivity within the central nervous system following antispastic epidural cervical spinal cord stimulation (SCS) treatment for patients with PSS. Following brief SCS treatment, the results showed improved functional connectivity between the anterior cingulate cortex (ACC) and the motor cortex of the contralateral hemisphere (Mayorova et al., 2023). Additionally, multiple case reports have identified that the damaged brain region involves the ACC in patients experiencing acute spasticity following stroke (Alzahrani et al., 2023; Alves et al., 2013). This suggests that the ACC may not only be involved in pain perception but also play a role in muscle stretch reflexes, with the underlying mechanisms requiring further investigation.

From a macro perspective, the three brain networks associated with pain processing are the Sensorimotor Network (SMN), the Salience Network (SN), and the Default Mode Network (DMN) (Damascelli et al., 2022). These three networks all influence the recovery of motor control and coordination abilities in stroke patients to varying degrees. Research indicates that the pain stimulus abnormally activates the SN, while SMN and DMN activation levels decrease. Furthermore, functional coupling between the central executive network and the SN is enhanced (Kim et al., 2018). Because the core brain regions of SN are the ACC and insula, it exhibits heightened sensitivity to pain perception (Li et al., 2024). This suggests that patients’ attention and brain resources are diverted to pain perception networks when they experience abnormal pain sensations. As a result, the SMN is disturbed. This disturbance causes abnormal motor control command output, which promotes the onset of spasms (Darvish et al., 2025). Pundik et al. discovered that spasticity severity was strongly connected with decreased sensory network activation after stroke, and that increased sensory network activation was associated with improved spasticity after treatment (Pundik et al., 2014). Additionally, the activity level of the DMN in stroke patients was significantly lower than that in healthy controls (Li et al., 2022), while increased synchronous functional connectivity between the SMN and DMN was beneficial for motor function recovery in stroke patients (Wu et al., 2020). Research suggests that acupuncture may facilitate effective connections between neural networks by regulating multiple networks and transmitting information via the DMN as a relay station, thereby improving PSS and cognitive impairment (Fu et al., 2017). In conclusion, pain acts as an exacerbating catalyst for spasticity in patients who have both pain and spasticity following a stroke. Therefore, pain management should not be ignored when treating spasticity. We hope that future research will unambiguously clarify the fundamental relationship between pain and spasticity, allowing for more targeted treatment.

## 6 Brain-gut axis and gut-muscle axis network

Theories like the gut-muscle axis and the brain-gut axis have been put forth one after the other as biotechnology continues to progress. Interestingly, there is a connection between these two hypotheses and PSS (Figure 7).

**Figure 7:**
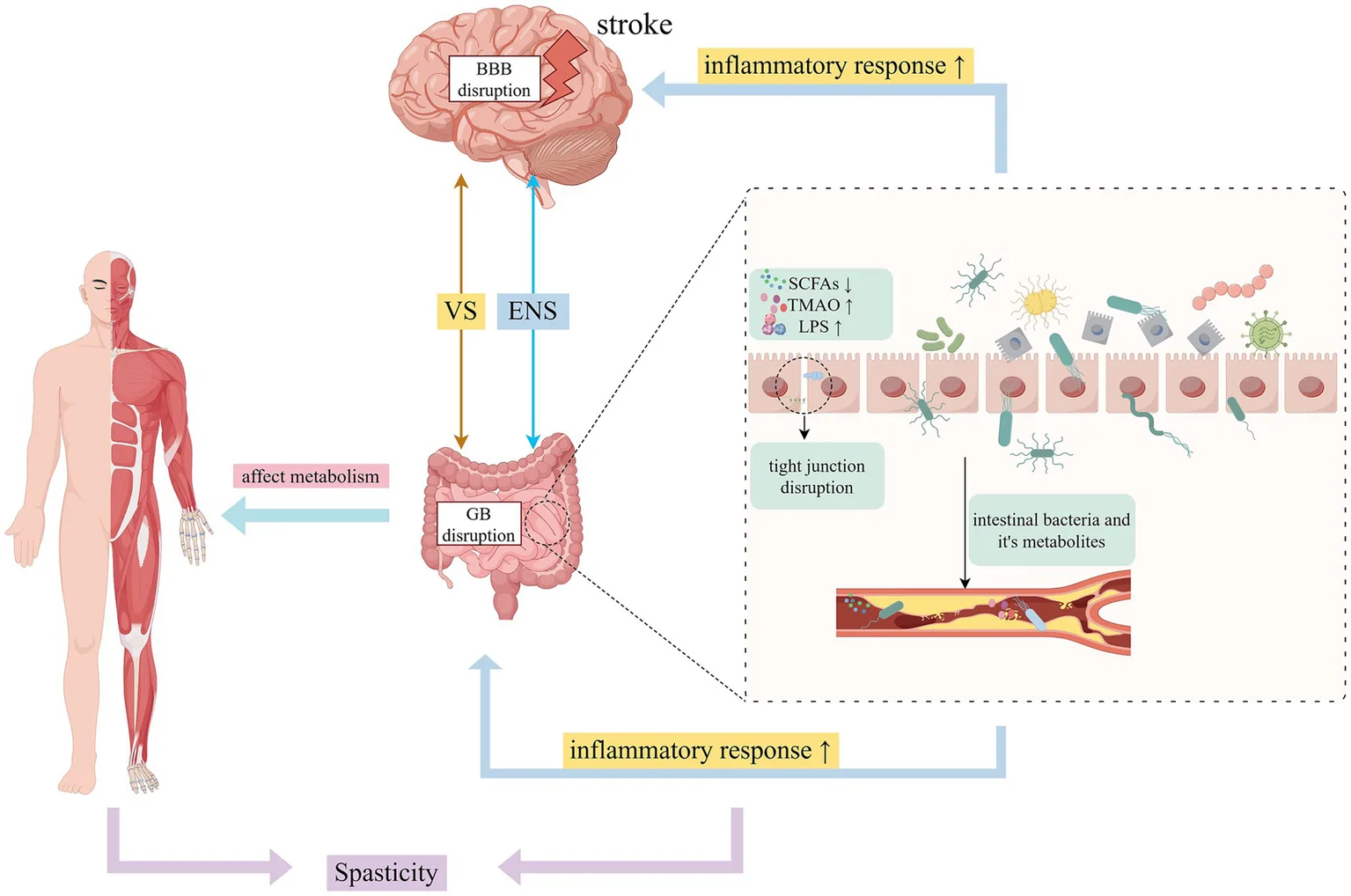
The relationship between the Brain-Gut Axis and Gut-Muscle Axis and post-stroke spasticity. Brain-Gut Axis, Post-stroke gut microbiota dysbiosis triggers a series of pro-inflammatory responses within the intestine, which in turn amplify inflammatory levels in the brain through a bottom-up mechanism, thereby establishing the environmental foundation for spasticity development. Gut-Muscle Axis, Gut macrobiotic symbiosis also impacts the metabolic state of peripheral muscles, directly influencing the structural basis for spasticity occurrence. BBB, Blood brain barrier; GB, Gut barrier; VS, Vagus system; ENS, Enteric nervous system; SCFAs, Short chain fatty acids; TMAO, Trimethylamine N-oxide; LPS, Lipopolysaccharide. This picture was drawn by Figdraw.

The brain-gut axis is a complex network facilitating bidirectional communication between the brain and the gut, primarily achieved through multiple pathways including the enteric nervous system, the vagus nerve, the endocrine system, and the immune system (Sorboni et al., 2022). In the past, PSS was not commonly linked to the gut-brain axis. However, recent research suggests that the gut-brain axis may promote spasticity development by exacerbating the inflammatory response following a stroke (Sun et al., 2024). From a fundamental perspective, pathological mechanisms such as excitotoxicity, oxidative stress, and inflammatory responses following stroke constitute the essential causes of subsequent functional impairments, including spasticity. The brain-gut axis plays a crucial role in exacerbating these inflammatory responses. The gut microbiota and its metabolites play a central role in the brain-gut axis network. Research shows that patients with ischemic stroke have an overabundance of Enterobacteriaceae during the acute and subacute stages. Through the lipopolysaccharide (LPS)-Toll-like receptor-4 pathway, this may quickly cause systemic inflammation, worsening cerebral infarction (Xu et al., 2021).

Another gut microbiota metabolite, trimethylamine N-oxide (TMAO), directly increases the risk of thrombosis. The risk of stroke is substantially positively connected with elevated TMAO levels (Liu et al., 2023). Moreover, TMAO can penetrate the blood–brain barrier(BBB), which can lead to oxidative stress and inflammatory reactions (Tu and Xia, 2024). Furthermore, the gut microbiota’s primary metabolic product, short-chain fatty acids (SCFAs), promotes the formation of tight junctions to lessen damage to the epithelial barrier. In stark contrast to the effects of LPS and TMAO, this increases the integrity of the BBB and the intestinal mucosal barrier, lowers inflammation, and improves prognosis (Dalile et al., 2019; Ahmed et al., 2022). Studies have shown that post-stroke gut microbiota dysbiosis leads to a reduction in short-chain fatty acids (such as butyrate), compromises the integrity of the blood–brain barrier, and exacerbates neuroinflammation (Wang et al., 2025). Nonetheless, research has shown that acute stroke patients have lower levels of SCFAs in their feces and bacterial abundance that produces SCFAs (Tan et al., 2021). Additionally, the reduction in Lactobacillus following a stroke may disrupt GABA synthesis, which could impair neuroprotective mechanisms (Henry et al., 2021).

According to clinical research, stroke patients’ lower limb spasticity decreases significantly, and their recovery of lower limb motor function is aided by abdominal moxibustion therapy, which is founded on the brain-gut axis theory and treats the brain through the gut (Zhuo et al., 2025). Studies on animals showed that rats with PSS had considerably higher serum TMAO levels and lower quantities of SCFAs. Pretreatment with electroacupuncture enhanced the content of SCFAs, reduced TMAO levels, and alleviated spasticity (Sun et al., 2024).

According to recent research, the gut-muscle axis can facilitate interactions between the gut microbiota and skeletal muscle (Morella et al., 2023). Skeletal muscle is thought to be the structural basis of PSS, and changes in the gut microbiota are thought to affect the metabolism and health of skeletal muscle. A novel perspective based on the gut-muscle axis was put out by Han et al. They thought that the recovery of PSS patients’ balance and posture depended heavily on the strength of their core muscles, and that abdominal moxibustion could control the intestinal flora disorder, strengthen the core muscles through the gut-muscle axis, and subsequently help with spasm (Han et al., 2024). However, the underlying mechanisms remain unclear. It should be mentioned, nonetheless, that there is currently a dearth of thorough research on the connection between PSS and the gut muscle axis through both clinical and animal trials. It can only be considered a speculative hypothesis at this time. It is envisaged that in-depth investigation would be carried out in subsequent studies.

New therapeutic approaches for PSS can be explored based on the gut-brain axis and gut-muscle axis networks. To enhance gut microbial diversity and control the dynamic balance of the gut microbiota, for example, fecal microbiota transplantation and exogenous probiotic supplements can be utilized (Zhou et al., 2023; Benakis and Liesz, 2022; Zeng et al., 2019; Chen et al., 2019). Regulating brain and skeletal muscle function from the gut and starting from the bottom up and working inward may be a direction worthy of further investigation.

## 7 Treatment methods

Neurostimulation and medication are now the main clinical treatments for PSS. We have reached the following conclusions based on the network processes spanning the five levels mentioned above and considering new therapeutic strategies, see Table 2 for a summary.

**Table 2:** Summary of emerging therapeutics for PSS.

Therapy type	Intervention	Target mechanism	Current clinical outcomes
Pharmacotherapy	Baclofen (oral/ITB)	Central nervous system network (spinal level); Neurotransmitter network (GABAergic); Myofascial network (Ach)	Oral baclofen: improves generalized spasticity; ITB: significantly alleviates pain, enhances quality of life.
	BoNT-A		improves proprioception, and breaks the pain-spasticity cycle.
Neuromodulation	Spinal cord stimulation	Central nervous system network (spinal and cortical levels)	Spasticity relief; enhanced functional connectivity between brainstem and premotor cortex.
	Vagus nerve stimulation	Gut-brain axis network (anti-inflammatory); Central nervous system network (cerebello-thalamo-cortical network)	Combined with rehabilitation training: relieves hand and wrist spasticity, improves upper limb motor function; current evidence remains limited.
Regenerative medicine	Stem cell therapy	Central nervous system network (neural remodeling); Gut-brain axis (intestinal barrier repair); Neurotransmitter network (GABAergic)	Improved FMA scores; enhanced interhemispheric and ipsilateral connectivity; case reports: improvement in both spasticity and aphasia. Most studies remain at experimental stage, with limited evidence specific to PSS.
Traditional Chinese medicine treatment	Acupuncture and Chinese herbal formula	Central nervous system network; Neurotransmitter network (GABAergic)	Significant reduction in MAS scores; Improvement in limb motor function;

ITB, intrathecal baclofen; Ach, acetylcholine; FMA, Fugl-Meyer assessment; PSS, post-stroke spasticity.

### 7.1 Pharmacological treatment

Baclofen and botulinum toxin type A are currently the most widely used drugs in the clinical treatment of PSS.

The most widely used oral antispasticity drug in clinical practice at the moment is baclofen, a GABA receptor agonist that mainly acts on the spinal cord to produce its antispasticity effects. Baclofen operates on GABA-B receptors on the presynaptic membranes of layers I–IV of the spinal cord (Saulino, 2018), and the medial RST plays a dominating role at the spinal cord level in individuals with PSS, according to the previously discussed central nervous system and neurotransmitter networks. It controls the imbalance between excitation and inhibition at the spinal cord level by inhibiting voltage-gated calcium channels and activating potassium channels through a G-protein-coupled mechanism. This reduces calcium influx and suppresses the excessive release of excitatory neurotransmitters like glutamate. However, because of the mode of delivery, GABA-B receptors in the brain may unavoidably become activated, which could have negative effects on the central nervous system like weariness and cognitive decline. Therefore, intrathecal baclofen (ITB) may be recommended for stroke patients who have significant generalized hypertonia. According to research by Michael Creamer et al., ITB can considerably reduce pain, enhance quality of life, and lessen spasms in people with PSS when compared to oral antispasmodic drugs. According to the study, as was covered in the part on the pain network previously in this article, PSS patients’ quality of life should be improved by addressing both spasticity and pain (Creamer et al., 2018). The study shows that both spasticity and pain are objectives that must be given equal consideration when aiming to improve the quality of life of PSS patients, even though the small sample size makes it difficult to determine whether the pain relief obtained through ITB in PSS patients is related to improvements in spasticity.

The most effective treatment for focal spasticity is type A botulinum toxin. Increased muscle tone results from the excessive release of acetylcholine (Ach) at the nerve endings of the motor end-plates in muscles with myofascial trigger points, according to the previously provided knowledge on the myofascial network. In order to stop exosomes from fusing with the presynaptic membrane, botulinum toxin type A specifically cleaves synaptosomal-associated protein 25 (SNAP-25) on receptors on the presynaptic membrane at the neuromuscular junction. This decreases spasm, prevents signals from being transmitted from α-motor neurons to muscle fibers, and inhibits the production of acetylcholine (Ach) (Zhao and Yang, 2026). According to recent research, peripheral BoNT-A injections can also cause the central nervous system to undergo adaptive remodeling. According to research, BoNT-A can modify the excitation-inhibition balance at the spinal cord level by lowering spindle sensitivity and aberrant sensory input to Class Ia afferent neurons (Hok et al., 2021). After receiving a BoNT-A injection, patients’ proprioception gradually returns to normal as their muscle stiffness gets better. The functional reorganization of cortical networks is also driven by this normalization of peripheral sensory input. Imaging studies show that important brain regions, such as the sensorimotor cortex, cerebellum, and posterior parietal cortex, exhibit notable alterations in patterns of activity and functional connectivity after BoNT-A treatment (Veverka et al., 2023; Vinehout et al., 2021), and that these alterations aid in the recovery of patients’ motor function. Furthermore, some BoNT-A molecules are transported retrogradely via axoplasmic transport to the spinal cord’s dorsal horn, where they prevent the release of pain mediators like glutamate and substance P by obstructing the fusion of protruding vesicles with the presynaptic membrane (Rasetti-Escargueil and Palea, 2024). This effectively ends the vicious cycle of spasm-pain-spasm.

### 7.2 Spinal cord stimulation

Spinal cord stimulation(SCS) is a minimally invasive neuromodulation technique that involves implanting electrodes into the epidural space of the spinal cord or near peripheral nerves. These electrodes deliver microcurrents of specific frequencies to the spinal cord. Parameters can be adjusted according to different therapeutic objectives to modulate the function of neural circuits. SCS is commonly known for treating pain (Widman et al., 2025), but its benefits extend far beyond that. SCS can also be used to treat motor dysfunction caused by spinal cord injury (Angeli et al., 2025), alleviate pain (Petersen et al., 2025), facilitate arousal in vegetative states (Piedade et al., 2023), improve rigidity and gait abnormalities associated with Parkinson’s disease (Gao et al., 2020), and address spasticity resulting from stroke. The first study using cervical SCS at the C3-T1 segment for upper limb functional rehabilitation after stroke has been reported by Marc P. Powell and his colleagues. The findings showed that sustained stimulation enhanced functional movement and upper limb muscular strength without causing any major side effects (Powell et al., 2023). According to another functional magnetic resonance imaging (fMRI)-based study, eight patients with brain injuries showed a significant improvement in spasticity and an approximate 42% increase in functional connectivity between the brainstem and the right premotor cortex after receiving cervical SCS (Mayorova et al., 2023). According to a 2024 systematic analysis that included 34 studies, SCS significantly improved brain injury-related spasticity compared to the control group, with a 68% subjective improvement rate (Jung et al., 2024). By improving presynaptic inhibition, inhibiting excessive stimulation of Ia afferents, and altering spinal cord networks, SCS may reduce spasms by decreasing aberrant muscle tone and lessening the intensity of stretch reflexes. It may also facilitate the reorganization of central neural networks and the restoration of normal motor control patterns through rhythmic stimulation with carefully calibrated parameters.

### 7.3 Vagus nerve stimulation

Vagus nerve stimulation (VNS) is a novel neuromodulation technique that delivers electrical signals to the vagus nerve. It has been approved by the U. S. Food and Drug Administration for the treatment of epilepsy, depression, headaches, and ischemic stroke (Afra et al., 2021; Liu et al., 2022; Andalib et al., 2023). The vagus nerve contains afferent fibers (80%) and efferent fibers (20%), enabling it to regulate autonomic nervous functions and behaviors in organs such as the gastrointestinal tract, as well as higher brain regions, including the prefrontal cortex, limbic system, and parietal cortex (Andalib et al., 2023; Vargas-Caballero et al., 2022). Given the vagus nerve’s potent neuromodulatory capabilities, numerous international clinical studies have explored the use of VNS for treating ischemic stroke, yielding favorable outcomes (Dawson et al., 2021; Dawson et al., 2016; Kimberley et al., 2018). Based on the above discussion of the gut-brain axis, we know that the inflammatory response triggered by stroke is a major factor contributing to spasticity. The vagus nerve has strong anti-inflammatory properties; it stimulates immune cells like macrophages to release acetylcholine by activating the cholinergic anti-inflammatory pathway. Acetylcholine binds to α7 nicotinic acetylcholine receptors (α7nAChR), inhibits the NF-κB signaling pathway, and consequently lowers the release of pro-inflammatory cytokines like TNF-α, IL-6, and IL-1β (Jelinek et al., 2024). Interestingly, multiple studies have found that VNS also demonstrates promising therapeutic effects for PSS. research on animals has shown that VNS promotes synaptic plasticity by activating the brain-derived neurotrophic factor (BDNF)/cAMP/PKA /phospho-cAMP -responsive element-binding factor (CREB) pathway (Li et al., 2020). Zhang et al. recruited 36 stroke patients with hemiplegia. The experimental group received upper limb rehabilitation robotics combined with transcutaneous auricular vagus nerve stimulation (taVNS), while the control group received upper limb rehabilitation robotics combined with sham taVNS stimulation. After 3 weeks of treatment, the experimental group demonstrated significantly greater reduction in hand and wrist spasticity compared to the control group (Chang et al., 2021). Additionally, research has demonstrated that VNS enhances recovery from ischemic stroke by boosting tissue repair through neurotrophic factors and decreasing neuroinflammation by transforming microglia from a pro-inflammatory (M1) to a neuroprotective (M2) phenotype. In order to promote functional recovery following stroke through anti-inflammatory effects, improved stability of the cerebellar-thalamic-cortical pathway, and promotion of neural remodeling, Wang et al.’s study creatively combines the powerful immunomodulatory effects of VNS with the excitotoxicity-suppressive effects of fastigial nucleus stimulation (FNS) (Wang et al., 2025). Thus, according to the preceding description of gut-brain axis networks and central nervous system networks, VNS not only enhances the microenvironment and has anti-inflammatory effects, but it also modifies cortical neural networks, all of which contribute to functional recovery. Furthermore, it is crucial to remember that in order to improve functional recovery and reduce the likelihood of negative reactions, stroke patients receiving VNS therapy need to be thoroughly screened. Even though there is now very little clinical evidence to support vagus nerve stimulation as a treatment for PSS, it is still a potentially effective supplementary therapeutic strategy. There may be a deeper underlying relationship because the vagus nerve’s afferent fibers mostly project to the nucleus of the solitary tract, which has intimate fiber connections with the medullary reticular development tissue that is very significant in the pathophysiology of PSS (Proietti et al., 2025). More thorough research is needed to understand how vagus nerve stimulation and rehabilitative training improve spasticity.

### 7.4 Stem cell therapy

Stem cell therapy has demonstrated enormous promise in the treatment of stroke as regenerative medicine continues to progress. According to research, the gut-brain axis, neural remodeling, and the control of central nervous system networks may all be connected to stem cell therapy’s capacity to support functional recovery following a stroke.

Due to stem cells’ capacity for self-renewal, tissue homing, and multisystem differentiation (Lin et al., 2017), stem cell transplantation for the treatment of stroke has steadily grown in popularity as a study topic. The primary stem cells used for treating stroke are neural stem cells and mesenchymal stem cells (MSCs) (Li et al., 2021). Endogenous neural stem cells can improve brain injury through neuronal replacement, remyelination, and secretion of neurotrophic factors. However, their clinical application is limited by complex factors such as apoptosis, uncontrolled differentiation, and an unfavorable microenvironment (Liu et al., 2022). Because of their distinct immunomodulatory properties, MSCs have gained attention in stem cell transplantation research as a means of reducing inflammatory reactions after stroke. MSCs are widely available and can be administered through diverse routes (Zhang et al., 2022).

The following pathways are now thought to be the main ways that MCS improves the prognosis for stroke patients: (1) Immunomodulatory effects mitigate inflammation. White blood cells accumulate extensively in the injured area after stroke. Overactivated microglia express a phagocytic phenotype and secrete large amounts of pro-inflammatory cytokines such as Interleukin-1β, Interleukin-6 and Tumor Necrosis Factor-α, leading to cell death (Li et al., 2021). Anti-inflammatory factors such as interleukin-4, interleukin-10, and tumor necrosis factor-β are secreted increasingly after MCSs transplantation, while expression of pro-inflammatory factors decreases (Zhang et al., 2022). Furthermore, MCS-secreted CXC3CL1 can cause microglia to express a neuroprotective phenotype, which has neuroprotective effects (Chen et al., 2023). (2) Secreting Neurotrophic Factors. MCSs can release or stimulate the release of neurotrophic factors such as BDNF and glial cell line-derived neurotrophic factor as well as growth factors including nerve growth factor, vascular endothelial growth factor, and platelet-derived growth factor. These neurotrophic factors promote neurogenesis and angiogenesis, decrease the extent of cerebral infarction, and lessen cell death. Research shows that transplanting MCSs with BDNF and GDNF genes performs better than native MCSs in terms of increasing neurological function and decreasing the size of infarcts (Zhang et al., 2022). (3) Paracrine Effects. Research has demonstrated that MCSs can secrete extracellular vesicles (EVs), which exert beneficial effects such as reducing neuroinflammation, promoting neurogenesis, and facilitating angiogenesis (Kim et al., 2020). Compared to MCSs transplantation, EVs offer advantages such as the ability to cross the blood–brain barrier and reduce the risk of cell-associated infarction (Li et al., 2023a; Zhang et al., 2021). As bioengineering and clinical practice have become increasingly integrated in recent years, a meta-analysis has shown that engineered EVs are more therapeutically effective than natural EVs in the treatment of ischemic stroke (Li et al., 2023b). In addition, MCSs promote functional recovery after stroke by inducing angiogenesis and promoting neurogenesis.

According to recent study, MCS may enhance the microenvironment by lowering inflammation and encouraging the release of neurotrophic factors in addition to the three immune-modulating methods already discussed. These effects include protecting the function of residual tissue in damaged brain regions, promoting the reorganization of central nervous system networks, regulating the GABA network to correct imbalances in the excitatory-inhibitory state, creating favorable conditions for neural functional remodeling, and facilitating the recovery of motor function.

In recent years, multiple studies have demonstrated that MCSs transplantation exhibits positive efficacy in promoting motor function recovery following stroke (Jaillard et al., 2020; Chung et al., 2021; Lee et al., 2022). Multiple animal studies have demonstrated that MCSs exert neuroprotective effects through mechanisms including inhibiting ischemia-induced microglial activation, enhancing the expression of neurotrophic factors such as BDNF, reducing glial scar formation, and alleviating neuroinflammation (Li et al., 2021; Oshita et al., 2020; Yabuno et al., 2023; Noh et al., 2020; Lee et al., 2020). In clinical trials, some studies have also demonstrated that MCSs promote functional recovery after stroke. Results from Jungsoo et al.’s randomized controlled trial(RCT) on MCSs treatment for cerebral infarction showed significantly higher improvement rates in Fugl-Meyer scores in the MCSs group compared to the control group. Interhemispheric connectivity and ipsilateral connectivity were also significantly increased in the MCSs group. Furthermore, the study revealed that stem cells can protect the corticospinal tract from degeneration, thereby promoting functional recovery (Lee et al., 2022). In another RCT assessing autologous MSCs therapy for subacute cerebral infarction, Jaillard et al. administered autologous bone marrow-derived MSCs intravenously to treat motor dysfunction after cerebral infarction. The treatment group’s NIHSS scores, Fugl-Meyer scores, and task-related fMRI activity showed notable improvements (Jaillard et al., 2020). Additionally, after administering human bone marrow-derived mesenchymal stem cells (hBM-MSCs) intravenously in a mouse model of ischemic stroke, Sammali et al. discovered that the density of PV^+^ GABAergic interneurons in the perilesional area had significantly increased after 5 weeks. This suggests that MSCs can protect vulnerable inhibitory interneurons after stroke and regulate the excitatory-inhibitory balance. More significantly, the percentage of PV^+^ cells surrounded by the perineuronal network (PNN) was decreased by MSC therapy. MSCs encourage neural remodeling in injured brain regions by lowering the fraction of the PNN, a specialized extracellular matrix component that limits structural plasticity and stabilizes established synaptic connections (Sammali et al., 2017).

Although there is currently little research on treating PSS, Stancioiu et al. found that intravenous or intrathecal administration of CD271 + stem cells alleviates spasticity and improves aphasia in patients with chronic stroke and is a safe therapeutic approach (Stancioiu et al., 2020).

Furthermore, stem cell therapy may facilitate functional recovery following a stroke via the gut-brain axis, according to certain research. Su et al. discovered that the intestinal mucus layer thins after an acute ischemic stroke, which causes bacterial translocation and LPS entrance into the circulation. This shift is explained by goblet cells’ reduction of mucin production as a result of increased systemic glucocorticoid levels. By boosting PPARγ signaling in hepatocytes, bone marrow-derived mesenchymal stem cells encourage glucocorticoid catabolism by upregulating HSD11B2 and SRD5A1 expression and downregulating HSD11B1 expression. This lowers systemic glucocorticoid (GC) levels and eventually restores mucin production in intestinal goblet cells. According to this study, stem cells can promote functional recovery by mending the intestinal barrier, which in turn reduces systemic inflammation (Su et al., 2025). Another study discovered that bone marrow-derived mesenchymal stem cell therapy altered the composition of the gut microbiome in mice with ischemic stroke, boosting the number of lactobacilli and bacteria that produce short-chain fatty acids (SCFAs) and facilitating neurological recovery (Zhao et al., 2021). Though it has a lot of promise, there is currently relatively little data on stem cell therapy for PSS. Future studies should clarify the underlying mechanisms and investigate if stem cells can be used as an adjuvant treatment for PSS.

Currently, stem cell transplantation for stroke treatment remains largely in the early stages of experimental research, with only a few studies having achieved clinical outcomes. Future research on stem cell transplantation for PSS should concentrate on carrying out more excellent clinical investigations. These studies should investigate optimal delivery routes and time windows, explore key factors influencing dosage, and ultimately determine the optimal therapeutic dose. At the same time, safety issues need to be given top priority, and any tumor growth stimulation needs to be well watched. Additionally, future approaches could integrate gene modification or stem cell pretreatment with transplantation, or combine stem cell transplantation with existing PSS treatment regimens to enhance therapeutic efficacy.

### 7.5 Traditional Chinese medicine treatment

A significant amount of evidence-based medical evidence has been gathered, and Traditional Chinese therapy therapies, including acupuncture and herbal therapy, are frequently employed in the clinical treatment of PSS in addition to the previously described neuromodulation and regenerative medicine techniques.

In acupuncture practice, the acupoints most commonly selected for the treatment of PSS are predominantly those of the Yangming meridians of the hand and foot, including Waiguan (TE5), Quchi (LI11), Shousanli (LI10), Zusanli (ST36), and Jianyu (LI15) (Chen et al., 2026). Acupuncture in conjunction with rehabilitation training has been proven in several recent trials to considerably lower MAS scores and enhance limb motor function (Xue et al., 2022; Wang et al., 2025; Ma and Li, 2025). According to pertinent research, acupuncture may alleviate PSS in a number of ways, such as by controlling the excitability of spinal α-motor neurons, encouraging the release of inhibitory neurotransmitters like glycine and GABA, enhancing local cerebral blood flow, and modifying the expression of neurotrophic factors (Ma and Li, 2025; Zhang et al., 2024; Kang et al., 2025).

PSS is frequently treated in clinical practice with traditional Chinese medicine formulae like Buyang Huanwu Decoction and Shaoyao Gancao Decoction (Zhang et al., 2022; Li and Wang, 2026; Zhang and Chen, 2023). Many clinical investigations conducted in the last few years have shown that traditional Chinese medicine is effective in treating PSS. Clinical studies and animal experiments have shown that traditional Chinese medicine reduces spasticity and has neuroprotective effects thru anti-inflammatory and antioxidant actions, inhibition of glutamate excitotoxicity, regulation of synaptic plasticity, and activation of multiple signaling pathways, including CCR5/CREB and Nogo-A/NgR (Zeng et al., 2022; Zhu et al., 2015; Wu et al., 2026; Sun et al., 2026).

It should be mentioned that there are still several limitations to the present research on the use of acupuncture and traditional Chinese medicine for the treatment of PSS, such as small sample sizes, a lack of standardized protocols, and challenges in conducting blinded investigations. High-quality, extensive, multicenter clinical trials are needed to confirm the effectiveness of these treatments. Additionally, the natural fusion of acupuncture and traditional Chinese medicine with contemporary neurostimulation technologies to create a step-by-step rehabilitation approach that combines Western and traditional Chinese medicine shows promise for offering PSS patients the best, customized treatment plans and is a path worth further investigation.

## 8 Conclusion

The pathogenesis of PSS is complex and likely mediated by multiple mechanisms, potentially involving various neural pathways in the central nervous system. It is also associated with abnormal changes in peripheral tissues, pain, and the gastrointestinal system, among other factors. New and developing therapeutic strategies for PSS, including SCS, VNS, and stem cell therapy, hold significant promise and potential. Therefore, future research should delve deeper into these avenues to enhance our comprehensive understanding of the pathological mechanisms underlying PSS, ultimately bringing hope to patients and their families.
