# Correction: Post-stroke spasticity: advances in network mechanisms and therapeutics

**DOI:** 10.3389/fnhum.2026.2035182

**Published:** 2026-10-29

**Authors:** Wei Yao, Yuanbin Yang, Zhiyi Wang, Longyue Yi, Deya Daneva, Zhuangwei Fang, Keyun Gan, Zhiqi Lin

**Affiliations:** 1 Department of First Rehabilitation Medicine, Wangjing Hospital of China Academy of Chinese Medical Sciences, Beijing, China; 2 School of Sports Medicine and Rehabilitation, Beijing Sport University, Beijing, China

**Keywords:** complication, network mechanisms, spasticity, stroke, therapeutics

An incorrect **Funding** statement was provided. The correct funder is [Scientific and Technological Innovation Project of China Academy of Chinese Medical Sciences]/the correct **Funding** statement reads:

[The author(s) declared that financial support was received for this work and/or its publication. This work was supported by Scientific and Technological Innovation Project of China Academy of Chinese Medical Sciences (CI2026A03014) and High level Chinese Medicine Hospital Construction Project of Wangjing Hospital of China Academy of Chinese Medical Sciences (WJYY-XZKT-2023-24). The funders had no role in study design, data collection, and analysis, decision to publish, or preparation of the manuscript].

The original version of this article has been updated.

